# Tolerance induction in memory CD4 T cells is partial and reversible

**DOI:** 10.1111/imm.13263

**Published:** 2020-10-27

**Authors:** Joshua I. Gray, Shaima Al‐Khabouri, Fraser Morton, Eric T. Clambey, Laurent Gapin, Jennifer L. Matsuda, John W. Kappler, Philippa Marrack, Paul Garside, Thomas D. Otto, Megan K.L. MacLeod

**Affiliations:** ^1^ Institute of Infection, Immunity and Inflammation University of Glasgow Glasgow UK; ^2^ Department of Anesthesiology University of Colorado Denver Aurora Colorado USA; ^3^ National Jewish Health Denver Colorado USA; ^4^ Present address: Columbia Center for Translational Immunology Columbia University New York New York USA

**Keywords:** memory CD4 T cells, tolerance, proliferation, mitotic catastrophe

## Abstract

Memory T cells respond rapidly in part because they are less reliant on a heightened levels of costimulatory molecules. This enables rapid control of secondary infecting pathogens but presents challenges to efforts to control or silence memory CD4 T cells, for example in antigen‐specific tolerance strategies for autoimmunity. We have examined the transcriptional and functional consequences of reactivating memory CD4 T cells in the absence of an adjuvant. We find that memory CD4 T cells generated by infection or immunisation survive secondary activation with antigen delivered without adjuvant, regardless of their location in secondary lymphoid organs or peripheral tissues. These cells were, however, functionally altered following a tertiary immunisation with antigen and adjuvant, proliferating poorly but maintaining their ability to produce inflammatory cytokines. Transcriptional and cell cycle analysis of these memory CD4 T cells suggests they are unable to commit fully to cell division potentially because of low expression of DNA repair enzymes. In contrast, these memory CD4 T cells could proliferate following tertiary reactivation by viral re‐infection. These data indicate that antigen‐specific tolerogenic strategies must examine multiple parameters of Tcell function, and provide insight into the molecular mechanisms that may lead to deletional tolerance of memory CD4 T cells.

## Graphical abstract

We have examined the response of memory CD4 T cells to tolerogenic signals. Although memory CD4 T cells could respond to antigen delivered without adjuvant, they undergo cell death upon further restimulation. Our data suggest that the Tcells die as a consequence of mitotic catastrophe that occurs when cells are unable to complete cell division.

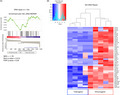

**Abbreviations**
BAC: bacterial artificial chromosome
DEG: differently expressed genes
Dox: doxycycline
IAV: influenza A virus
IFNγ: interferon‐gamma
IL: interleukin
i.n.: intranasal
MedLN: mediastinal lymph node
OVA: ovalbumin
TCR: T‐cell receptor
TNF: tumour necrosis factor
TRACE: T‐cell reporter of activation and cell enumeration
Treg: regulatory T cell

## Introduction

Memory CD4 T cells play central roles in enhancing immune protection against pathogens the host has previously encountered.^1^ However, activated and memory CD4 T cells also contribute to disease processes in chronic inflammatory conditions, including rheumatoid arthritis and multiple sclerosis.^2^, ^3^, ^4^ Most current treatments for these conditions require continued use of drugs that dampen or deplete immune mediators or cells. A cure for these diseases will require deletion or retraining of the CD4 T cells that contribute to pathology.

Antigen‐specific tolerance strategies have been used for many years to treat allergies, and there are ongoing trials in autoimmune patients.^5^, ^6^, ^7^, ^8^ The underlying rationale for these strategies is based on our knowledge of tolerance induction in T cells, mainly developed from experiments examining Tcell receptor (TCR) activation of naïve CD4 T cells in the absence of costimulatory and inflammatory signals.^9^, ^10^, ^11^ Much less is known about the consequences of activating memory CD4 T cells through their TCR alone. Memory CD4 T cells can respond more quickly to a secondary challenge. This is, in part, because they are less reliant on a heightened level of costimulatory signals,^12^, ^13^, ^14^ although memory CD4 T‐cell cytokine responses do require the presence of costimulatory molecules.^14^, ^15^ While this contributes to rapid pathogen control, this presents significant hurdles for treatments that aim to induce antigen‐specific tolerance in autoimmunity, allergy or transplantation.^16^, ^17^

A deeper understanding of the functional and molecular consequences of activating memory CD4 T cells with TCR signals alone is required to surmount these hurdles. We recently demonstrated that memory CD4 T cells reactivated with antigen delivered in the absence of adjuvant return to the memory pool and survive long term.^18^ However, tertiary reactivation led to a curtailed response. Here, we address two outstanding questions: 1. whether the consequences of reactivating memory CD4 T cells with antigen alone are similar in lymphoid organs and peripheral tissues and 2. what the underlying cellular changes responsible for the curtailed tertiary response are. These are important questions as the pathology for many autoimmune and allergic conditions is present in peripheral tissues, and understanding the mechanisms of memory CD4 Tcell tolerance is essential to improve treatments and monitor therapeutic success. Our data show that while memory CD4 Tcell responses are altered following exposure to tolerogenic signals, their ability to respond and produce inflammatory cytokines is not permanently restrained.

## Materials and methods

### Animals

10‐week‐old female C57BL/6 mice were purchased from Envigo (UK). Tcell reporter of activation and cell enumeration (TRACE) and C57BL/6 mice were maintained at the University of Glasgow under specific pathogen‐free conditions in accordance with UK home office regulations (Project Licence P2F28B003) and approved by the local ethics committee.

To generate mice in which rtTA reports interleukin‐2 (IL‐2) expression, we used recombineering to extract the upstream 8.389 kb section of the IL‐2 promoter from a Bacterial Artificial Chromosome (BAC) RP24208L3 (BAC resource at Children's Hospital Oakland Research Institute, Buffalo, New York). This was subcloned into a plasmid containing the human CD2 locus control region and linked to the rtTA sequence. The transgene, cut and purified from the construct backbone, was used to create transgenic mice at the Transgenic mouse facility at National Jewish Health in Jackson Lab C57BL/6 animals. Two founder pups were identified by PCR, but only one was fertile. Progeny of this animal were bred with B6.Cg‐Tg(tetO‐cre)1Jaw/J (006234) and B6.129X1‐Gt(ROSA)26Sor^tm1(EYFP+)Cos^ (006148) both from Jackson Laboratories.

### Immunisations and infections

For influenza A virus (IAV) studies, C57BL/6 mice were briefly anesthetized using inhaled isoflurane and infected with 200–300 plaque forming units of IAV strain A/WSN/33 in 20 μl of PBS intranasally (i.n.). IAV was prepared and tittered in MDCK cells. Infected mice were rechallenged with 100PFU of X31 (kindly provided by Prof James Stewart, University of Liverpool). Infected mice were weighed daily. Any animals that lost more than 20% of their starting weight were humanely euthanized. TRACE mice were given Dox+chow (Envigo) for a total of 7 days starting 2 days prior to immunisation with 40 µg of ovalbumin (OVA) protein (Worthington) conjugated to 20 µm polyethylene carboxylate beads (Polysciences Inc.) with 20 µg of polyinosinic:polycytidinic acid (InvivoGen) and 20 µg of anti‐CD40 (BioXcell) s.c in the scruff. Recipients of TRACE EYFP+T cells were given 40 µg of OVA with/out 10 µg lipopolysaccharide (LPS) i.v. in 100 µl of PBS. NP_311–325_ was conjugated to OVA using Imject Maleimide‐activated OVA according to the manufacturer's instruction (Thermo Fisher). Briefly, the OVA was reconstituted with 200 µl of Ultrapure water. 200 µl PBS was added to 2 mg of NP_311–325_ peptide (JPT) and combined with the OVA and incubated at room temperature for 2 hours. Excess peptide was removed by centrifugation using a 30 K centricon filter (Sigma) for 15 minutes at 3500 g with three washes with PBS. The conjugate was filter sterilized and the concentration measured using a Nanodrop (Thermo Fisher). Mice were immunized with 1 μg NP_311–325_‐OVA delivered i.p with 0.1 mg alum. For mitosis analysis, C57BL/6 mice were immunized with 20 μg NP_311–325_ peptide (JPT) with 10 μg of LPS i.v. After 30 days, they were re‐immunized with 20 μg NP_311–325_ peptide with/out 10 μg LPS followed 30 days later by i.p. immunisation with 5 μg NP‐OVA with 0.1 mg of alum i.p.

### FACS sorting and cell transfers

TRACE mice were euthanized 8 days post‐immunisation, and single‐cell suspensions were prepared. Lymphoid organs, including spleen, mediastinal, axillary, brachial and mesenteric lymph nodes, from individual mice were pooled and pre‐enriched for CD4 T cells using EasySep^TM^ Mouse T Cell Isolation Kit (Stemcell Technologies). Live, single, EYFP+ CD4 T cells negative for MHCII, B220, CD8 and F4/80 were sorted on a BD FACS Aria. Sorted cells were washed in PBS and 100,000 cells transferred i.v. into naïve C57BL/6 mice.

### Tissue preparation

Mice were euthanized either by cervical dislocation or with a rising concentration of carbon dioxide and perfused with PBS‐5 mM EDTA in experiments examining lungs. Spleen and mediastinal lymph nodes (MedLNs) were processed by mechanical disruption. Single‐cell suspensions of lungs were prepared by digestion with 1 mg/ml collagenase and DNAse (Sigma) for 40 minutes at 37°C. Red blood cells were lysed from the spleen and lungs using lysis buffer (Thermo Fisher).

### Flow cytometry

A single‐cell suspension was stained with PE‐labelled IA^b^/NP_311–325_ (NIH tetramer core) at 37°C, 5% CO_2_ for 2 hours in complete RMPI (RPMI with 10% foetal calf serum, 100 μg/ml penicillin‐streptomycin and 2 mM l‐glutamine) containing Fc block (24G2). Surface antibodies were added, and the cells incubated for a further 20 minutes at 4°C. Antibodies used were as follows: anti‐CD4 BUV805 (BD Biosciences; clone: RM4‐5) or CD4 APC‐Alexa Fluor 780 (eBioscience; RM4‐5), anti‐CD44 BUV395 (BD Biosciences; clone: IM7), anti‐CXCR5 BV785 (BioLegend; clone:L138D7), anti‐PD‐1 BV711 (BioLegend: 29F.1A12) and ‘dump’ antibodies: B220 (RA3‐6B2), anti‐CD8 (53‐6.7) and MHC II (M5114) all on eFluor‐450 (eBioscience). Cells were stained with a fixable viability dye eFluor 506 (eBioscience). In some cases, cells were then fixed with FoxP3 Transcription Factor Fixative kit (Thermo Fisher, UK) and stained with anti‐FoxP3 PeCy7 (eBioscience; FJK‐16S), anti‐Bcl2 FITC (Biolegend; Blc/10C4) and anti‐Ki67 BV605 (Biolegend; 16A8). Phosphorylated H3 was detected in cells fixed with 2%PFA/0.5% saponin using Alexa 647‐labelled anti‐Histone H3 (pS28) (HTA28, Thermo Fisher). Cells were acquired on a BD LSR or Fortessa and analysed using FlowJo (version 10 Treestar).

### Tcell cytokine analysis

Bone marrow‐derived dendritic cells were cultured as described^19^ in complete RPMI supplemented with X‐63 supernatant for 7 days. A single‐cell suspension was incubated with 10 μg/ml NP_311–325_ peptide for 2 hours prior to co‐culture with lungs, spleen or lymph node cells in complete RMPI at a ratio of approximately 10 T cells to 1 DC in the presence of Golgi Plug (BD Bioscience). Co‐cultures were incubated at 37°C, 5% CO_2_ for 6 hours. Cells were incubated with Fc block and surface stained with anti‐CD4 BUV805 (BD Biosciences; clone: RM4‐5) or CD4 APC‐Alexa Fluor 780 (eBioscience; RM4‐5), anti‐CD44 BUV395 (BD Biosciences; clone: IM7) and ‘dump’ antibodies: B220 (clone: RA3‐6B2), CD8 (53‐6.7) and MHC II (clone: M5114) all on eFluor‐450 (eBioscience). Cells were fixed with cytofix/cytoperm (BD Bioscience) for 20 minutes at 4°C and stained in perm/wash buffer with anti‐cytokine antibodies for 1 hour at room temperature (anti‐IFN‐γ PE (XMG1.2), anti‐TNF Alexa Fluor‐488 (MP6‐XT22) anti‐IL‐2 APC (JES6‐5H4) all from eBioscience.

### RNA isolation for RNA‐seq

CD4^+^ EYFP^+^ cells from the spleen, mediastinal, axillary, brachial and mesenteric lymph nodes of TRACE cell recipients were FACS sorted as above, and cell pellets were stored at −20°C prior to RNA extraction. RNA was extracted and purified from single‐cell suspensions using RNeasy Micro Kit (Qiagen) according to the manufacturer's instructions.

### RNA analysis

Sequencing and library prep were conducted by LCSciences Ltd. Total RNA was extracted using Trizol reagent (Invitrogen, CA, USA). Total RNA quantity and purity were analysed using a Bioanalyzer 2100 and RNA 6000 Nano LabChip Kit (Agilent, CA, USA), and all samples had RIN numbers >7.0. Approximately 10 µg of total RNA was subjected to isolate Poly (A) mRNA with poly‐T oligoattached magnetic beads (Invitrogen). Following purification, the poly(A)‐ or poly(A)+ RNA fractions were fragmented using divalent cations under elevated temperature. The cleaved RNA fragments were reverse‐transcribed to create the final cDNA library in accordance with the protocol for the mRNA‐Seq sample preparation kit (Illumina, San Diego, USA). The average insert size for the paired‐end libraries was 300 bp (±50 bp). Paired‐end sequencing was done on an Illumina Hiseq 4000 (lc‐bio, China). Cutadapt software^20^ was used to remove low‐quality reads and adaptor sequences. High‐quality reads were then mapped to a C57BL/6 mouse reference genome to which the EYFP sequence was added using HISAT2.^21^ Readcounts were obtained from bam files with FeatureCounts using the default parameters. Differently expressed genes (DEGs) were obtained with DESeq2, using RStudio (RStudio Inc). DEGs were visualized with a volcano plot using the ‘Enhancedvolcano’ package within R. DEGs with a fold change of at least 3 and q‐values less than *p* = 0.05 were classed as statistically significant. Heatmaps were generated using the heatmap2 function, using the DESeq2 normalized counts and Panther^22^ used to determine GO biological processes. GSEA analysis was conducted using UC San Diego/Broad Institute's GSEA software.^23^, ^24^

## ELISA

Ovalbumin‐specific antibody ELISAs were carried out as described.^18^ Serum from immunized and control mice was titrated in 2‐fold serial dilution on plates coated with OVA protein. Anti‐mouse IgG, IgG1 or IgG2c biotin detection antibodies (Thermo Fisher, UK) were used with Extravidin‐peroxidase (Sigma‐Aldrich) and SureBlueTMB substrate (KPL). Absorbance was measured at 450 nm using a Sunrise Absorbance Reader (Tecan). The absorbance of each sample was normalized to a positive control on each plate after the background absorbance from a blank well had been removed.

### Statistical analysis

Data were analysed using Prism version 7 software (GraphPad). Differences between groups were analysed by unpaired ANOVAs or T‐tests as indicated in figure legends. In all figures, * represents a *p* value of <0.05; **p > 0.01, ***p > 0.001 and ****p > 0.0001.

## Results

### Antigen‐specific memory CD4 T cells in lymphoid organs and in peripheral tissues respond to tolerogenic signals but fail to increase in number

To investigate the consequence of reactivating memory CD4 T cells with tolerogenic signals (antigen delivered without adjuvant), we needed to track antigen‐specific CD4 T cells following secondary and tertiary reactivation. To achieve this, we generated memory CD4 T cells in lymphoid organs and peripheral tissues by infecting C57BL/6 mice with WSN IAV intranasally (i.n.). We used MHC class II tetramers containing the immunodominant IAV peptide nucleoprotein (NP)_311–325_ to identify NP_311–325_‐specific memory CD4 T cells in the spleen, lung draining MedLN and lung (Figure S1).

Previously,^18^ we delivered antigen intravenously (i.v.) as it is a well‐established tolerogenic injection route.^18^, ^25^, ^26^, ^27^ As expected, i.v. injection of NP_311–325_ and the adjuvant PolyIC led to an increase of T cells within the spleen in mice previously infected with IAV. However, there was no increase in the numbers of antigen‐specific CD4 T cells in the MedLN or lung (Figure S2). These data suggested that the antigen failed to access the MedLN and lung and that this delivery route could not be used to address our questions.

**Figure 1:**
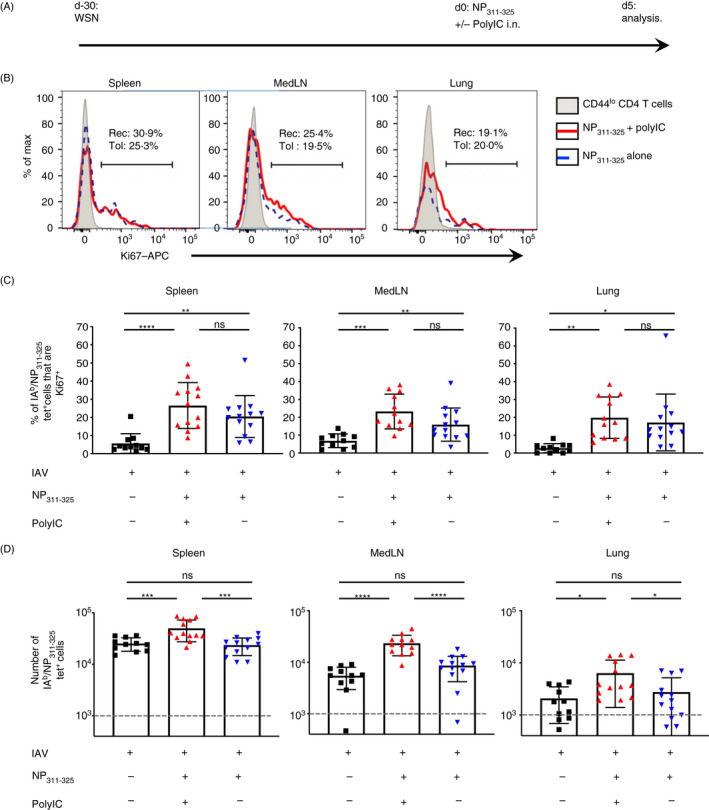
NP_311–325_‐specific memory CD4 T cells reactivated with peptide delivered in the absence of adjuvant respond but fail to accumulate. C57BL/6 mice were infected with influenza A virus (IAV) on day −30. On day 0, some of these mice were immunized with NP_311–325_, +/−, PolyIC i.n. IA^b^/NP_311–325_ CD44^hi^ CD4 T cells were examined 5 days later in the spleen, MedLN, and lung (A) and their Ki67 expression (B, C) or their numbers determined (D). In C and D, each symbol represents one mouse and error bars are SD. In D, the grey dashed line represents the background staining in naïve animals. Data are combined from 3 experiments (3–5 mice/experiment). Cells are gated as shown in Figure S1. All statistics calculated using a one‐way ANOVA with multiple comparisons; ns = not significant, *<0.05, **<0.01, ***<0.001, ****<0.0001.

In contrast, i.n. instillation of peptide in the presence (immunogenic) or absence (tolerogenic) of PolyIC led to reactivation of the memory CD4 T cells in all three organs (Figure 1A,B with representative FACS plots shown in Figure S3). Importantly, delivery of peptide in the absence of adjuvant i.n. to naïve animals led to functional tolerance of the antigen‐specific CD4 T‐cell population, validating this injection route for assessment of memory CD4 Tcell tolerance induction (Figure S4).

We first examined the immediate consequences of reactivating memory CD4 T cells with immunogenic or tolerogenic signals. Five days following instillation of NP_311–325_ peptide delivered with or without PolyIC, antigen‐specific CD4 T cells showed evidence of activation via increased expression of the proliferation marker, Ki67, in all three organs (Figure 1B,C). Memory CD4 T cells reactivated in the presence of adjuvant increased in number as expected (Figure 1D). In contrast, there was no accumulation of memory CD4 T cells reactivated following the tolerogenic instillation of peptide alone. This suggests that while these cells entered the cell cycle, they either failed to complete mitosis or rapidly underwent cell death following proliferation.

### Memory CD4 T cells previously exposed to tolerogenic signals fail to expand upon subsequent reactivation despite entry into the cell cycle

**Figure 2:**
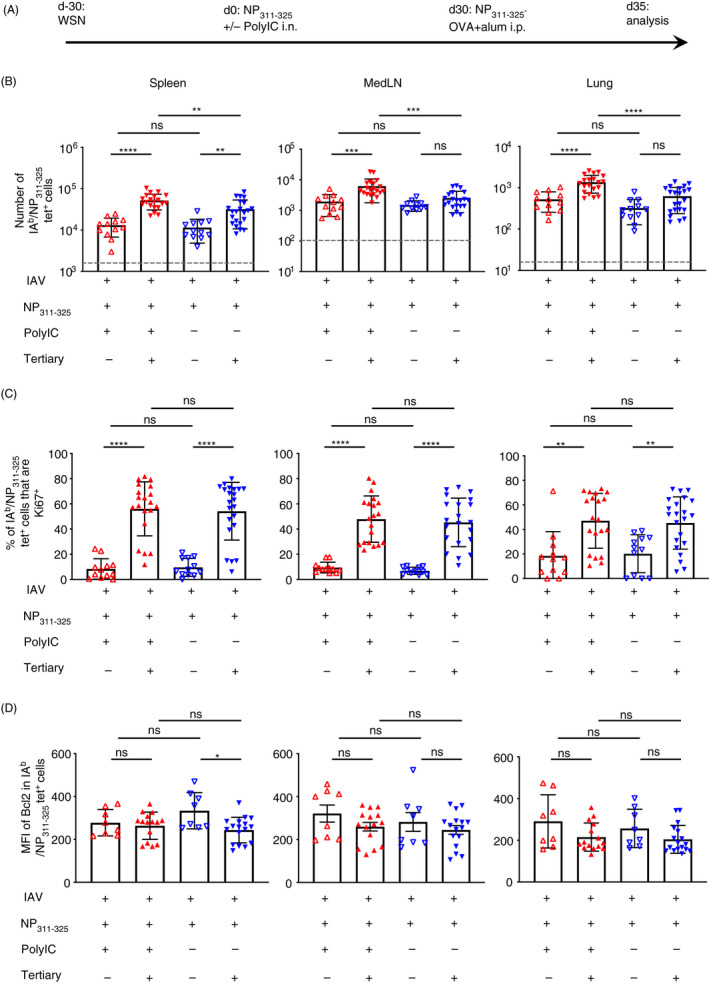
NP_311–325_‐specific memory CD4 T cells previously reactivated with peptide without adjuvant fail to accumulate in the lungs after tertiary reactivation. C57BL/6 mice were infected with influenza A virus (IAV) on day −30. On day 0, mice received NP_311–325_ +/− PolyIC and some of these mice were immunized i.p with NP‐OVA with alum on day 30 (A). The numbers of IA^b^/NP_311–325_ CD44^hi^ CD4 T cells were examined 5 days later in the spleen, MedLN, and lung (B) and their expression of Ki67 (C) and Bcl2 (D) determined. Each symbol represents one mouse, and error bars are SD. In B, the grey dashed line represents the background staining in naïve animals. Data are combined from 4 experiments (4–8 mice/experiment). All statistics calculated using a one‐way ANOVA with multiple comparisons; ns = not significant, *<0.05, **<0.01, ***<0.001, ****<0.0001.

To examine the longer term consequences of reactivating memory CD4 T cells with tolerogenic signals, we set up the experiment displayed in Figure 2A. Thirty days after infection with IAV, animals were given NP_311–325_ i.n. delivered with (immunogenic) or without (tolerogenic) PolyIC. After a further 30 days, we either examined the memory cells or performed a tertiary immunisation with NP_311–325_ conjugated to ovalbumin (OVA) protein delivered with the adjuvant alum.

Prior to tertiary reactivation, there were similar numbers of memory antigen‐specific CD4 T cells in the two groups in each organ (Figure 2B). The antigen‐specific CD4 T cells reactivated with an immunogenic secondary injection were able to mount a robust response in all organs upon tertiary reactivation. In contrast, the CD4 T cells previously exposed to tolerogenic signals expanded only slightly in the spleen and not at all in the MedLN and lung. There was, however, no difference in the expression of Ki67 in the reactivated CD4 T cells (Figure 2C).

We also examined the expression of the pro‐survival molecule, Bcl2, to determine whether the memory CD4 T cells were more prone to apoptosis following tolerogenic activation (Figure 2D with representative plots in Figure S5). However, there were no differences in the expression of Bcl2 between the two groups regardless of whether we examined the memory or the recalled cells in any of the three organs. This suggests increased apoptosis could not account for the poor accumulation of the tertiary reactivated memory CD4 T cells exposed to tolerogenic signals.

### Memory CD4 T cells reactivated with tolerogenic signals are not converted to regulatory T cells

**Figure 3:**
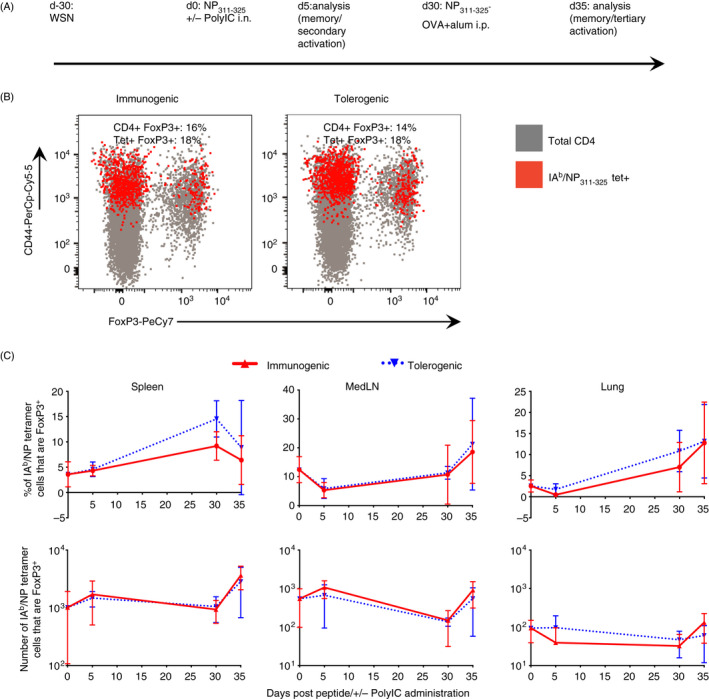
NP_311–325_‐specific memory CD4 T cells exposed to soluble antigen in the absence of adjuvant are not converted to Tregs. C57BL/6 mice were infected with influenza A virus on day −30. On day 0, mice received NP_311–325_ +/− PolyIC i.n. and some of these mice were immunized i.p with NP‐OVA and alum on day 30 (A). The expression of FoxP3 by IA^b^/NP_311–325_ CD44^hi^ CD4 T cells was determined and shown in red and total CD4 T cells in grey in representative FACS plots (B) and the percentages and numbers of FoxP3 by IA^b^/NP_311–325_ CD44^hi^ CD4 T cells days 0, 5 and 35 shown in (C). The data are from 1 experiment/time‐point with 4–5 mice/group/time‐point. Each symbol represents the mean of between 4 and 5 mice, and the error bars are SD, ***<0.001.

Previous studies have shown that activation with antigen alone can lead to tolerance via the induction of FoxP3‐expressing regulatory T cells.^28^ We found few NP_311–325_‐specific FoxP3^+^ cells at any time‐point examined (Figure 3). While there was a slightly increased percentage of antigen‐specific FoxP3+ cells in the mice exposed to tolerogenic signals at one time‐point in the spleen, there were no differences in the number of FoxP3+ NP_311–325_‐specific cells between the groups. This suggests that regulatory T cell (Treg) conversion does not explain the poor accumulation of antigen‐specific memory CD4 T cells previously exposed to tolerogenic signals.

### Memory CD4 T cells exposed to tolerogenic signals display evidence of mitotic catastrophe following reactivation

Thus far, our experiments indicated that memory CD4 T cells activated with tolerogenic signals can survive in the memory pool but accumulate poorly upon tertiary reactivation. To take an unbiased approach to investigate this failure, we performed transcriptomics analysis. For this, we required a significant number of memory CD4 T cells that could be easily isolated for analysis. As the identification of antigen‐specific CD4 T cells by MHC tetramers requires ligation of the TCR by MHC molecules and the number of epitope‐specific cells are limited, we developed a novel triple transgenic reporter mouse, TRACE. We generated a transgenic animal in which the IL‐2 promoter drives the expression of rtTA. These animals were crossed to B6.Cg‐Tg(tetO‐cre)1Jaw/J mice and B6.129X1‐Gt(ROSA)26SorTm(EYFP+)Cos mice. In these animals, T cells activated through the TCR when the animals are given doxycycline (Dox) become permanently EYFP+ (Figure S6A).

Feeding of the Dox^+^ diet for 1 week was sufficient to induce a small population of EYFP^+^ cells, even in the absence of immunisation. However, delivery of OVA conjugated to 20 μm polyethylene carboxylate beads in combination with the strong adjuvant combination of anti‐CD40 and PolyIC^29^ drove an increased population of EYFP^+^ CD4 T cells above background (Figure S6B,C). OVA was used for these studies as we required a protein containing multiple CD4 Tcell epitopes that could be obtained free of contaminating microbial products, which would be present in recombinant IAV proteins. Moreover, we found that recombinant nucleoprotein had intrinsic adjuvant properties.^30^

**Figure 4:**
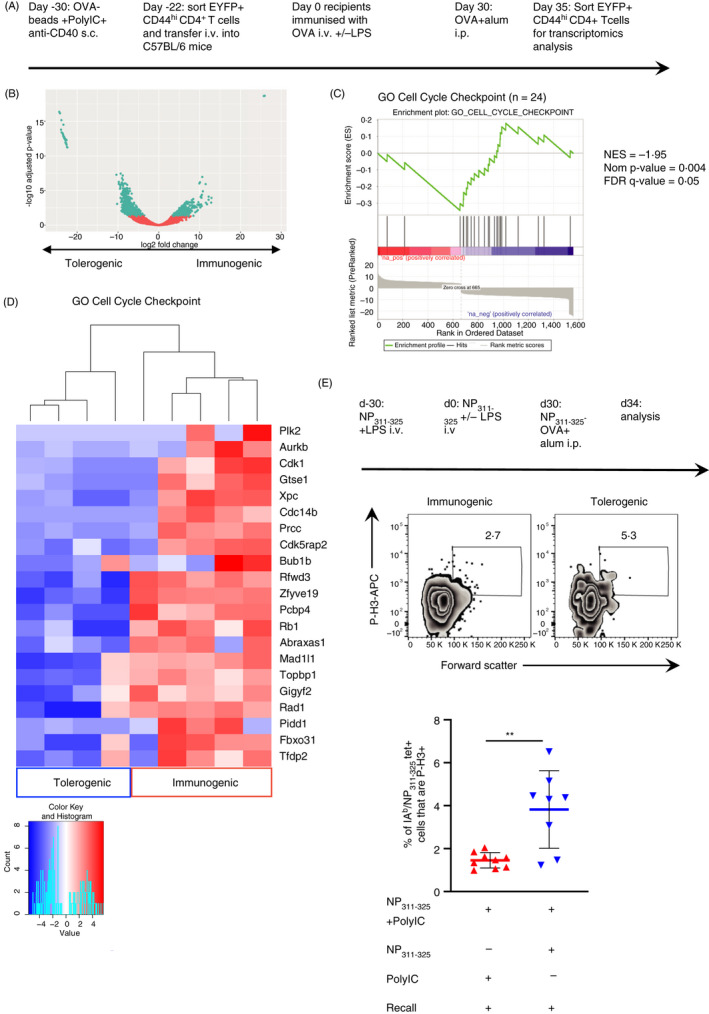
Transcriptomics and cell cycle analysis indicate that memory CD4 T cells exposed to tolerising signals undergo mitotic catastrophe following further reactivation *in vivo*. FACS‐sorted EYFP^+^ CD4 T cells from TRACE mice immunized with OVA + anti‐CD40 and PolyIC were transferred into naïve C57BL/6 mice that were then immunized with OVA +/− LPS then re‐immunized with OVA + alum i.p. 30 days later (A). EYFP+ CD4 T cells were FACS sorted after a further 5 days and RNA isolated for transcriptomic analysis. The DEGs are displayed in a volcano plot (B). GESA and heatmap show the expression of DEGs contained within the GO term ‘Cell Cycle Checkpoints’ (GO: 0000075) (C). DEGs within the GO term and expressed at lower levels in the tolerogenic samples are displayed in a heatmap (D). C57BL/6 mice immunized with NP_311–325_ and LPS were injected with NP_311–325_ +/− LPS 30 days later and finally immunized after a further 30 days with NP‐OVA + alum. 5 days later the percentages of forward scatter high IA^b^/NP_311–325_ tetramer + cells that expressed phosphorylated Histone3 were examined (E). In (E), cells are gated as in Figure S1 and plots are concatenated from 4 mice per group. Data are combined from 2 experiment with 4–5 mice/group, and error bars are SD. Statistical analysis in B calculated by a *T*‐test, **<0.01.

To reactivate the memory CD4 T cells, we returned to systemic i.v. delivery of OVA protein as this is widely accepted as a consistent tolerogenic route.^18^, ^25^, ^26^, ^27^ We found that secondary immunisation with OVA in the TRACE mice led to anaphylactic shock, likely a consequence of anti‐OVA antibodies. To avoid this, we sorted EYFP^+^ CD4 T cells at day 8 after immunisation and transferred these cells into naïve C57BL/6 animals that were injected with OVA or OVA and LPS i.v. after 22 days and 30 days since the cells were first primed. Thirty days following this, the recipient animals were immunised with OVA^+^ alum and CD4^+^ EYFP^+^ cells isolated 5 days later for RNA‐seq analysis (Figure 4A).

Gene expression from 5 individual mice in each experimental condition was analysed. One sample from the tolerogenic group was excluded as the number of EYFP+ CD4 T cells collected was 2.5‐ to 10‐fold higher than any of the other samples, suggesting an abnormal response or potential contamination during sorting. Of the Differently Expressed Genes (DEGs), 898 were expressed at lower levels and 667 at higher levels in the tolerogenic group compared to the immunogenic group (Figure 4B). Analysis of DEGs expressed at higher levels in the tolerised samples failed to find consistent changes across all four samples. We, therefore, concentrated on DEGs that were expressed at lower levels in the tolerised samples. The top 33 DEGs expressed at lower levels that are represented on the top left of Figure 4B are listed in Table S1. Gene ontogeny (Panther^22^) analysis of the biological processes associated with these DEGs indicated overrepresentation of gene products involved in ‘DNA‐dependent DNA replication’, ‘spindle organization’ and ‘cell cycle checkpoints’ (Table 1 and File S1).

**Table 1:** Gene overrepresentation analysis of DEGs expressed at lower levels in EYFP+ CD4 T cells in tolerogenic groups (top 3).

	Number of genes	Number of genes within DEGs	Fold enrichment	Raw p value	Adjusted p value (FDR)
DNA‐dependent DNA replication (GO:0006261)	103	13	3.71	1.16E−04	3.98E−02
Spindle organization (GO:0007051)	134	15	3.29	1.22E−04	4.10E−02
Cell cycle checkpoint (GO:0000075)	152	16	3.1	1.41E−04	4.56E−02

We performed gene set enrichment analysis and found that the DEGs were enriched for genes within the GO term, Cell Cycle Checkpoints (Figure 4C); DEGs within this GO term that were expressed at lower levels in the tolerogenic samples are displayed as a heatmap (Figure 4D). A number of these molecules play key roles at various stages of the cell cycle and in spindle formation and function. These genes include the essential cyclin, Cdk1^31^; Aurkb, a key component of the Chromosome Passenger Complex required for normal spindle assemble^32^; Mad1l1 (also known as MAD1), a component of the spindle‐assembly checkpoint^33^, ^34^; and Cdk5rap2, which plays a number of roles in spindle checkpoints.^35^, ^36^

Dysfunction of the spindle checkpoint is linked to death by mitotic catastrophe, a form of cell death that occurs when cells are unable to complete mitosis.^37^, ^38^ As the percentages of CD4 T cells that were Ki67+ after tertiary activation were equivalent regardless of whether or not they had been activated with immunogenic or tolerogenic signals (Figure 2C), these data suggest that memory CD4 T cells activated with tolerogenic signals can enter the cell cycle but fail to complete cell division following tertiary reactivation. To investigate this, we examined the proportion of reactivated memory antigen‐specific T cells in mitosis reasoning that more CD4 T cells would be found in mitosis in reactivated cells previously exposed to tolerogenic signals as they would be ‘stuck’ in mitosis.

The percentages of reactivated antigen‐specific T cells in mitosis were determined by the expression of phosphorylated (p)Histone 3, present only during mitosis.^39^ We focused on cells with increased forward scatter as cells increase in size during cell division.^40^ CD4 T cells were examined 4 days after tertiary reactivation of mice first immunized with NP_311–325_ and LPS, then reactivated with NP_311–325_ delivered with or without LPS and finally reactivated with NP‐OVA+ alum (Figure 4E). Very few antigen‐specific T cells were positive for p‐H3, but consistently more CD4 T cells were p‐H3 positive in mice previously immunised with NP_311–325_ delivered without, than with, LPS (Figure 4E). This indicates that the cells in mice that received tolerogenic signals were more likely to be mitosis, suggestive of a failure to complete cell division.

**Figure 5:**
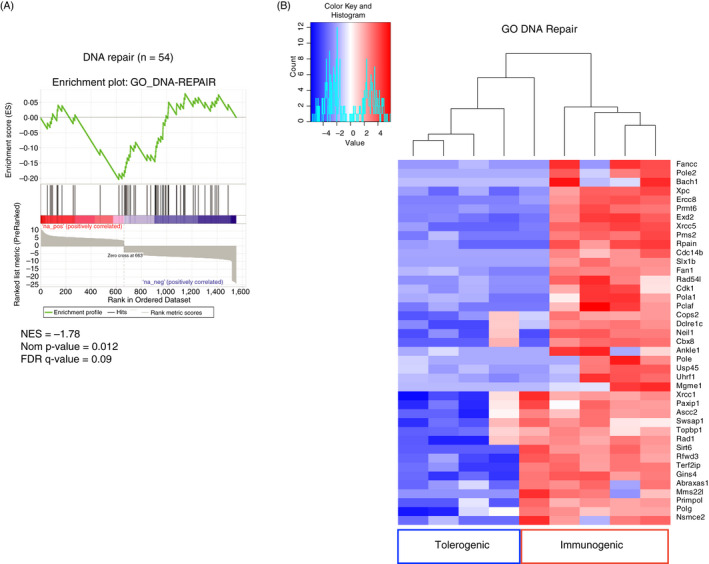
Transcriptomics analysis indicates that memory CD4 T cells exposed to tolerising signal have reduced expression of DNA repair enzymes. FACS‐sorted EYFP^+^ CD4 T cells from TRACE mice immunized with OVA+anti‐CD40 and PolyIC were transferred into naïve C57BL/6 mice that were then immunized with OVA +/− LPS then re‐immunized with OVA + alum i.p. 30 days later as in Figure 4. EYFP^+^ CD4 T cells were isolated by FACS after a further 5 days and RNA isolated for transcriptomic analysis. GSEA shows significant enrichment of the genes involved within the GO term ‘DNA repair’ (GO: 0006281) (A), and the DEGs expressed at lower levels in the tolerogenic samples are displayed in a heatmap (B).

Mitotic catastrophe often occurs in cells with DNA damage.^41^ We, therefore, examined whether any DEGs were enriched in genes involved in GO term DNA repair. This was indeed the case (Figure 5A); the DEGs expressed at lower levels in the tolerised samples are shown as a heatmap (Figure 5B). The GSEA of the DEGs expressed at lower levels in the tolerogenic samples indicates that multiple genes contribute to this enrichment. These data suggest that tolerised memory CD4 T cells display poor repair of their DNA during reactivation‐induced DNA synthesis and that this is a consequence of reduced expression of a number of different genes. This, coupled with the low expression of cell cycle checkpoint proteins, likely compromises their ability to commit to cell division.

### Memory CD4 T cells exposed to tolerogenic signals continue to produce cytokine but fail to provide accelerated help to primary responding B cells

Our data indicate that memory CD4 T cells reactivated with tolerogenic signals have impaired proliferative responses. We also wanted to determine whether these cells were impaired in other ways. To investigate this, we used the IAV infection model to generate sufficient cells in multiple organs to examine *ex vivo* cytokine production; cytokine responses are limited in antigen/adjuvant models.^42^

**Figure 6:**
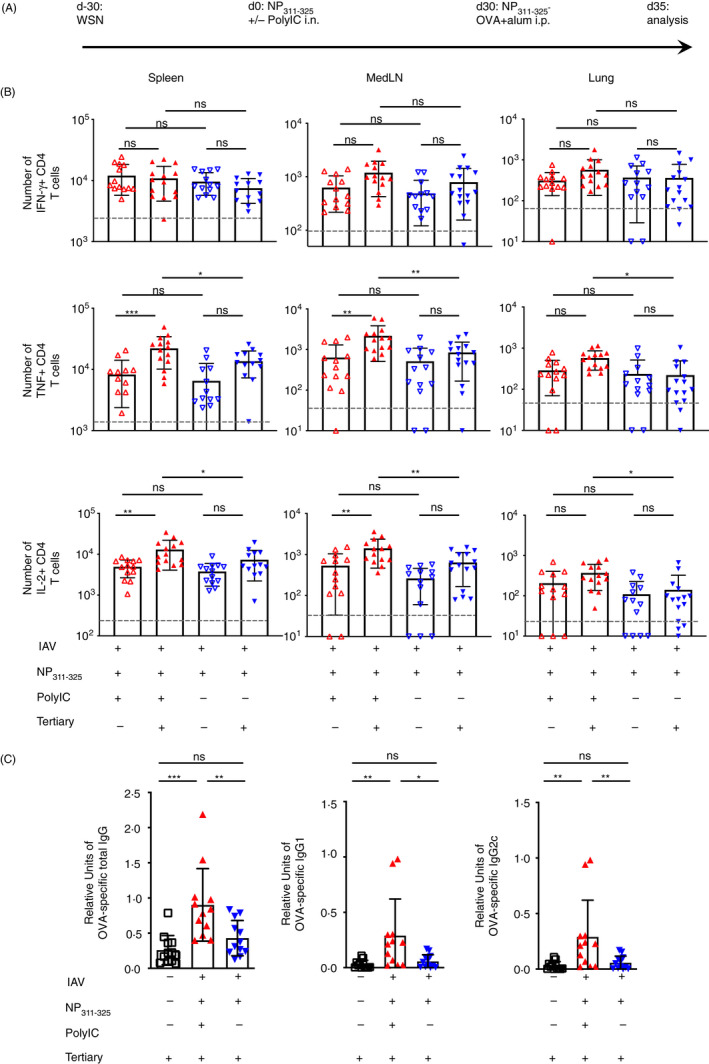
Activation of CD4 T cells with peptide in the absence of adjuvant does not affect CD4 Tcell cytokine production but does prevent them providing accelerated help to B cells. C57BL/6 mice were infected with influenza A virus (IAV) on day −30. On day 0, mice received NP_311–325_ +/− PolyIC and some of these mice were immunized i.p with NP‐OVA with alum on day 30 (A). On day 35, cells from the spleen, MedLN and lung were co‐cultured with bmDCs loaded with NP_311–325_ for 6 hours in the presence of Golgi Plug and the number of interferon‐gamma‐ (IFN‐γ), tumour necrosis factor‐ (TNF) and interleukin‐2 (IL‐2)‐producing CD44^hi^ CD4^+^ T cells examined (B). The levels of IgG, IgG1 and IgG2c anti‐OVA antibodies in the serum were determined on day 5 (C). Each symbol represents one mouse, and error bars are SD. In B, the grey dashed line represents the background staining in naïve animals. Data in B are combined from 2–3 experiments (3–5 mice/experiment). Data in C are combined from 3 experiments with 4 mice/experiment. All statistics calculated using a one‐way ANOVA with multiple comparisons; ns = not significant, *<0.05, **<0.01, ***<0.001, ****<0.0001.

Thirty days after mice were infected with IAV, they were injected with immunogenic or tolerogenic NP_311–325_ i.n. and then rested for 30 days (Figure 6A). Bone marrow dendritic cells loaded with NP_311–325_ were used to examine the *ex vivo* cytokine potential of the memory CD4 T cells and activated CD4 T cells from mice given a tertiary immunisation with NP_311–325_‐OVA and alum delivered i.p. (Figure S7).

The numbers of interferon‐gamma‐ (IFN‐γ), tumour necrosis factor‐ (TNF) or IL‐2‐producing antigen‐specific memory CD4 T cells were similar in mice exposed to immunogenic or tolerogenic NP_311–325_ peptide 35 days previously (Figure 6B) with no consistent changes found across the three organs and three cytokines. Five days after reactivation with NP‐OVA+alum, there was an increase of TNF and IL‐2 producing cells in the spleen and the MedLN in mice previously exposed to NP_311–325_ and PolyIC. In contrast, there was no increase in the number of cytokine‐producing cells in mice previously exposed to tolerogenic NP_311–325_. In neither group did we see an increase in IFN‐γ producing CD4 T cells. Together, these data suggest that, while exposure to tolerogenic signals affected accumulation of T cells, it did not prevent their ability to produce cytokines.

To investigate the functional responses of the T cells further, we examined their ability to provide accelerated help to primary responding OVA‐specific B cells.^18^, ^43^ We measured the levels of class‐switched, OVA‐specific antibody 5 days after the tertiary reactivation. As expected, primary responding mice had very little class‐switched, OVA‐specific antibody and IAV‐infected mice previously exposed to immunogenic signals had clearly detectable levels of OVA‐specific immunoglobulin.^43^ In contrast, IAV‐infected mice that had previously received tolerogenic signals failed to produce these antibodies at levels above primary immunised animals, demonstrating an impaired functional response (Figure 6C).

### Memory CD4 T cells exposed to tolerogenic signals expand following reactivation with influenza virus

**Figure 7:**
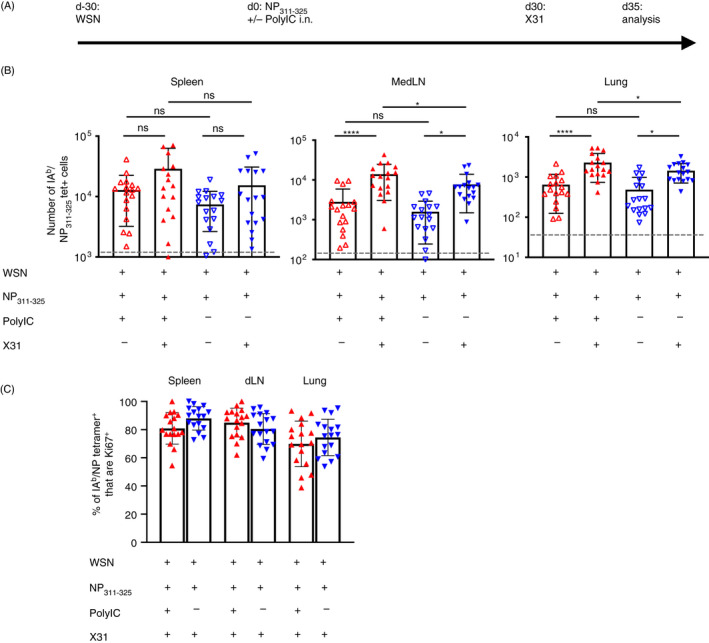
CD4 T cells exposed to peptide in the absence of adjuvant expand following re‐infection with influenza A virus (IAV). C57BL/6 mice were infected with IAV on day −30. On day 0, mice received NP_311–325_ +/− PolyIC i.n. and some of these mice were infected with 100PFU of X31 i.n. 30 days after this (A). The numbers of IA^b^/NP_311–325_ CD44^hi^ CD4 T cells were examined 5 days later in the spleen, MedLN, and lung (B) and their expression of Ki67 determined (C). In B and C, each symbol represents one mouse and error bars are SD. In A, the grey dashed line represents the background staining in naïve animals. Data are combined from 3 experiments (5–6 mice/experiment). All statistics calculated using a one‐way ANOVA with multiple comparisons; ns = not significant, *<0.05, **<0.01, ***<0.001, ****<0.0001.

Finally, we wanted to test whether the failure of CD4 T cells exposed to tolerogenic signals to accumulate could be rescued by reactivation with a more inflammatory stimulus. We therefore challenged IAV‐infected mice given immunogenic or tolerogenic signals with an heterosubtypic form of IAV, X31 (Figure 7A).

Five days following re‐infection, we found significant increases in the numbers of antigen‐specific CD4 T cells in the lungs and MedLN of mice regardless of their immunisation history (Figure 7B). This expansion was less clear in the spleen regardless of previous immunisation. In all organs, the majority of the antigen‐specific CD4 T cells were Ki67 positive indicating a more robust response following IAV infection compared to immunisation (Figure 7C versus Figure 2C).

## Discussion

Memory CD4 T cells respond to low doses of antigen and costimulatory signals, suggesting they will be refractory to tolerance induction.^12^, ^13^, ^14^, ^44^ This presents significant hurdles for therapies that aim to induce antigen‐specific Tcell tolerance.^6^, ^17^, ^45^

Our previous^18^ and current data demonstrate that some but not all functions of memory CD4 T cells are altered following the exposure of memory CD4 T cells to antigen delivered in the absence of adjuvant. Our findings demonstrate that tolerance induction in these cells is both subtle and complex. This contrasts with investigations of tolerance induction in naïve CD4 T cells that consistently show silencing of multiple Tcell functions.^9^, ^10^, ^11^, ^46^, ^47^

There have been inconsistent reports of whether memory CD8 T cells undergo tolerance induction following reactivation with antigen alone. Tolerance induction appears to be dependent on the specific location of the memory T cells with cells in lymphoid organs and the lung susceptible to deletion with exogenous antigen, while those in the central nervous system were resistant.^27^ In contrast, memory CD8 T cells essentially ignore and are therefore unaffected by antigen expressed through an inducible endogenous system.^48^ Thus, tolerance induction in memory CD8 T cells is highly dependent on the method of tolerogenic stimulation and the location of the cells.

Consistently in our own and others’ research, memory CD4 T cells reactivated with antigen alone fail to accumulate following tertiary activation with antigen and adjuvant.^18^, ^49^ Our analysis of cell proliferation and survival signals suggests that this is not a consequence of reduced entry into the cell cycle nor altered expression of anti‐apoptosis molecules. Instead, our data indicate that memory CD4 T cells reactivated following exposure to antigen fail to complete mitosis, a characteristic associated with the phenomenon of mitotic catastrophe.

Mitotic catastrophe has mainly been studied in tumour cells treated with ionizing radiation or drugs that cause DNA damage.^41^, ^50^, ^51^, ^52^ Our transcriptomic data indicate that reactivated memory CD4 T cells previously exposed to tolerogenic signals have reduced expression of molecules involved in sensing and repairing DNA damage and in the control of various stages of the cell cycle. Our data suggest, therefore, a novel form of cell death for memory CD4 T cells.

In contrast to the poor accumulation of memory CD4 T cells exposed to tolerogenic signals, this did not shut down cytokine production. We found only small increases in cytokine‐producing CD4 T cells following reactivation regardless of Tcell activation history. If these cells are not undergoing cell division, they would not be at risk of death via mitotic catastrophe. These data suggest that non‐cytokine‐producing memory CD4 T cells are more likely to proliferate than those committed to cytokine production. This agrees with the general concepts within the Tcentral/Teffector memory cell classification and with findings from ourselves and others that cells either making IFN‐γ, or that are CD62L^lo^, proliferate poorly on reactivation.^42^, ^53^, ^54^

We did find that memory CD4 T cells previously exposed to tolerogenic signals were unable to provide accelerated help for primary responding B cells, suggesting cell proliferation may be required for this functional response. This contrasts with our previous study in which memory cells exposed to tolerogenic signals could help primary responding B cells produce class‐switched antibody.^18^ There are multiple differences in experimental procedure between our previous study and the experiments here including the antigen (3 K peptide versus NP_311–325_) form of priming (antigen versus infection) and route of tolerance induction (intravenous versus intranasal) that could explain this difference. Regardless, in our studies and similar research from others, memory CD4 T cells reactivated with antigen delivered without adjuvant accumulate poorly following a subsequent immunisation.^18^, ^49^ This suggests that consistently and, regardless of specificity, priming, memory cell location and route of injection of tolerogenic signals, memory CD4 T cells reactivated with antigen in the absence of adjuvant proliferate poorly following reactivation with antigen and adjuvant.

Interestingly, we found that this poor accumulation could be rescued by re‐infection with IAV, a much more potent challenge to the host than immunisation. These data suggest that while the responses of memory CD4 T cells can be moderated by exposure to tolerogenic signals, these cells may not be permanently silenced. An alternative explanation is that a portion of the memory CD4 T cells are not reactivated by antigen immunisations and therefore remain blind to the tolerogenic signals and free to respond to the infection. Teasing apart these two possibilities will require detailed understanding of the micro‐location of memory CD4 T cells within peripheral and lymphoid organs and which antigen‐presenting cells reactivate memory CD4 T cells following immunisation and infection. A deeper understanding of these factors will be critical to address the most effective methods of antigen‐specific tolerance strategies. Our data, moreover, demonstrate the importance of analysing multiple phenotypic and functional parameters in trials of antigen‐specific therapy.^6^, ^45^

## Conflict of interest

The authors have no competing interests to declare.

## Author contribution

Joshua I. Gray designed and performed experiments, analysed data and wrote the manuscript; Thomas D. Otto and Shaima Al‐Khabouri analysed data; Fraser Morton, Eric T. Clambey, Laurent Gapin, Jennifer L. Matsuda, John W. Kappler and Philippa Marrack designed and produced essential tools; Paul Garside designed experiments; Megan K. L. MacLeod designed and performed the research, analysed data and wrote the manuscript. All authors approved the manuscript.

## Supporting information


**Figure S1.** Gating strategy for IAb/NP_311‐325_ tetramer+ CD44_hi_ cells.
**Figure S2.** Lung memory NP_311‐325_‐antigen‐specific CD4 T cells are not reactivated by antigen and adjuvant delivered i.v.
**Figure S3.** Identification of IAb/NP_311‐325_ tetramer+ cells in IAV memory mice reactivated with peptide delivered in the absence or presence of adjuvant.
**Figure S4.** Instillation of NP_311‐325_ peptide induces functional tolerance in naive animals.
**Figure S5.** Reactivated antigen specific memory CD4 T cells express similar levels of Bcl2 regardless of whether they were previously reactivated with peptide delivered with or without adjuvant.
**Figure S6.** TRACE mice enable identification of antigen‐reactive CD4 T cells.
**Figure S7.** Identification of NP_311‐325_ specific cytokine producing CD4 T cells.
**Table S1.** The top 33 gene.

**File S1.** Panther over‐representation test of GO terms for DEGs expressed at lower levels in tolerised memory CD4 T cells compared to control CD4 T cells.

## Data Availability

Transcriptomics data have been deposited on GEO, accession number GSE145310.
