# Supplementary material for: Tolerance induction in memory CD4 T cells is partial and reversible
Source: Immunology. 2020 Oct 27;162(1):68–83. doi: 10.1111/imm.13263 (PMC7730012; doi:10.1111/imm.13263)
Supplement: Supplementary file 1 — Figure S1. Gating strategy for IAb/NP311‐325 tetramer+ CD44hi cells. Figure S2. Lung memory NP311‐325‐antigen‐specific CD4 T cells are not reactivated by antigen and adjuvant delivered i.v. Figure S3. Identification of IAb/NP311‐325 tetramer+ cells in IAV memory mice reactivated with peptide delivered in the absence or presence of adjuvant. Figure S4. Instillation of NP311‐325 peptide induces functional tolerance in naive animals. Figure S5. Reactivated antigen specific memory CD4 T cells express similar levels of Bcl2 regardless of whether they were previously reactivated with peptide delivered with or without adjuvant. Figure S6. TRACE mice enable identification of antigen‐reactive CD4 T cells. Figure S7. Identification of NP311‐325 specific cytokine producing CD4 T cells. Table S1. The top 33 gene. [file IMM-162-68-s001.pdf]

Supplementary Figures, Gray et al

Supplementary Figure 1

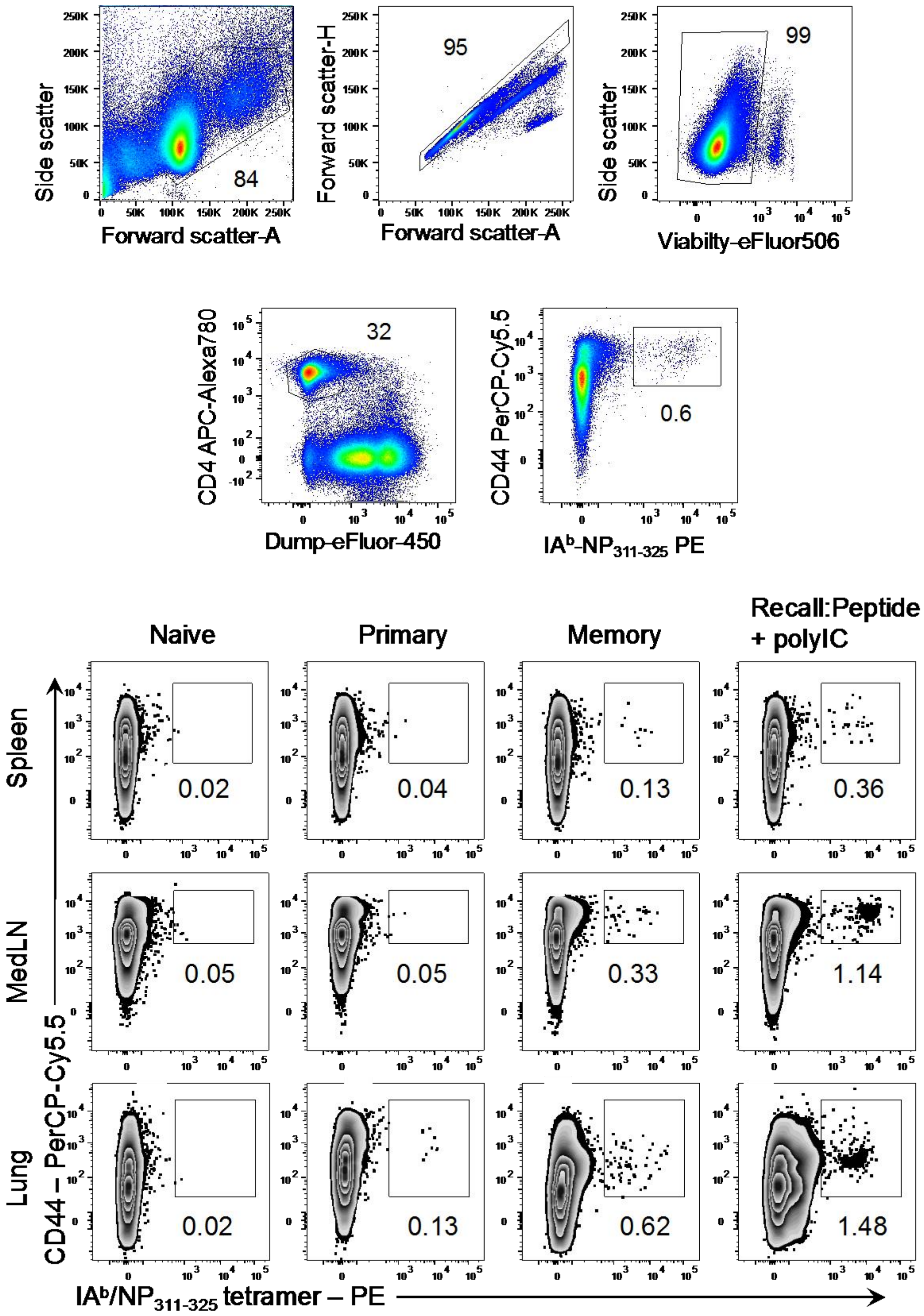

Supplementary Figure 1: Gating strategy for $IA_b/NP_{311-325}$ tetramer+ $CD44_{hi}$ cells

C57BL/6 mice were infected with IAV i.n. and some were given $NP_{311-325}$ peptide+PolyIC i.n. on day 30. The percentages of $IA_b/NP_{311-325}$ tetramer+ $CD44_{hi}$ CD4 T cells examined 35 days later in spleen, mediastinal LN, and lung. Cells are gated on live CD4+ lymphocytes that are negative for B220, F4/80, CD8, and MHCII+ as shown in the gating strategy. The numbers on the graph show the percentages of the cells present in the gate within each plot.

Supplementary Figure 2

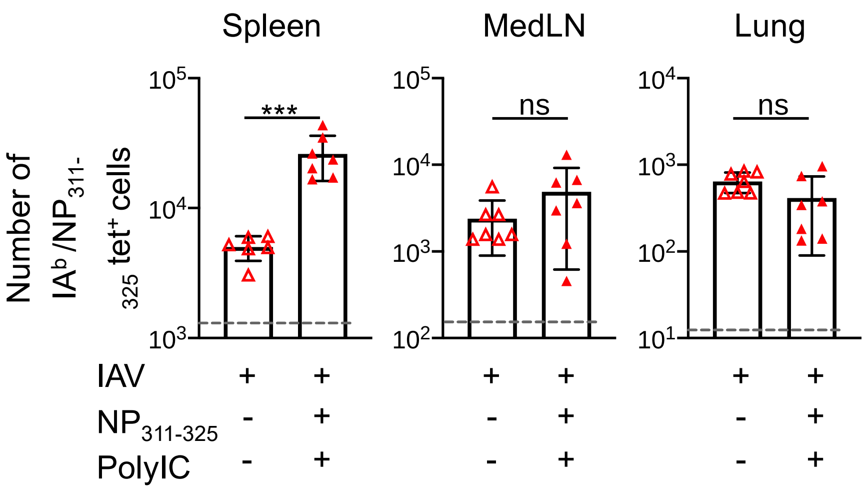

Supplementary Figure 2: Lung memory NP311-325-antigen-specific CD4 T cells are not reactivated by antigen and adjuvant delivered i.v.

C57BL/6 mice were infected with IAV i.n. on day -30. On day 0, some of these mice were immunised with NP311-325, + PolyIC intravenously. The numbers of IAb/NP311-325 CD44hi CD4 T cells were examined 5 days later in the spleen, MedLN, and lung. Each symbol represents one mouse and error bars are SD. The grey dashed line represents the background staining in naïve animals. Data are combined from two experiments (3-4mice/experiment). Statistics calculated using a one-way ANOVA with multiple comparisons; ns = not significant, * = <0.05, ** = <0.01, *** = <0.001, **** = <0.0001.

Supplementary Figure 3

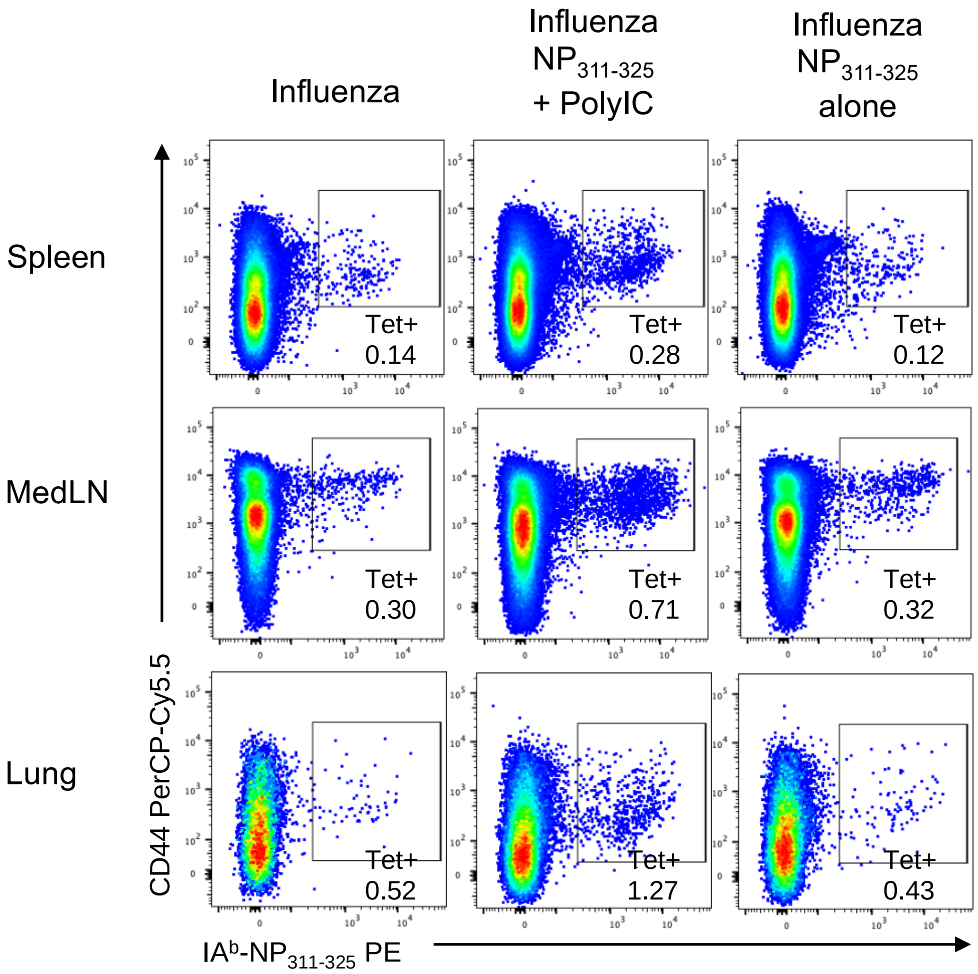

Supplementary Figure 3: Identification of IA_b/$NP_{311-325}$ tetramer+ cells in IAV memory mice reactivated with peptide delivered in the absence or presence of adjuvant

C57BL/6 mice were infected with IAV on day -30. On day 0, some of these mice were immunised with $NP_{311-325}$, +/-, PolyIC i.n. IA_b/$NP_{311-325}$ $CD44_{hi}$ CD4 T cells were examined 5 days later in the spleen, MedLN, and lung. Plots are representative of mice from 3 experiments (3-5mice/experiment). Cells are gated on CD4+ cells as shown in Supplementary Figure 1. Numbers in graphs show percentage of IA_b/$NP_{311-325}$ tetramer+ cells out of CD4 cells within the indicated gate.

Supplementary Figure 4

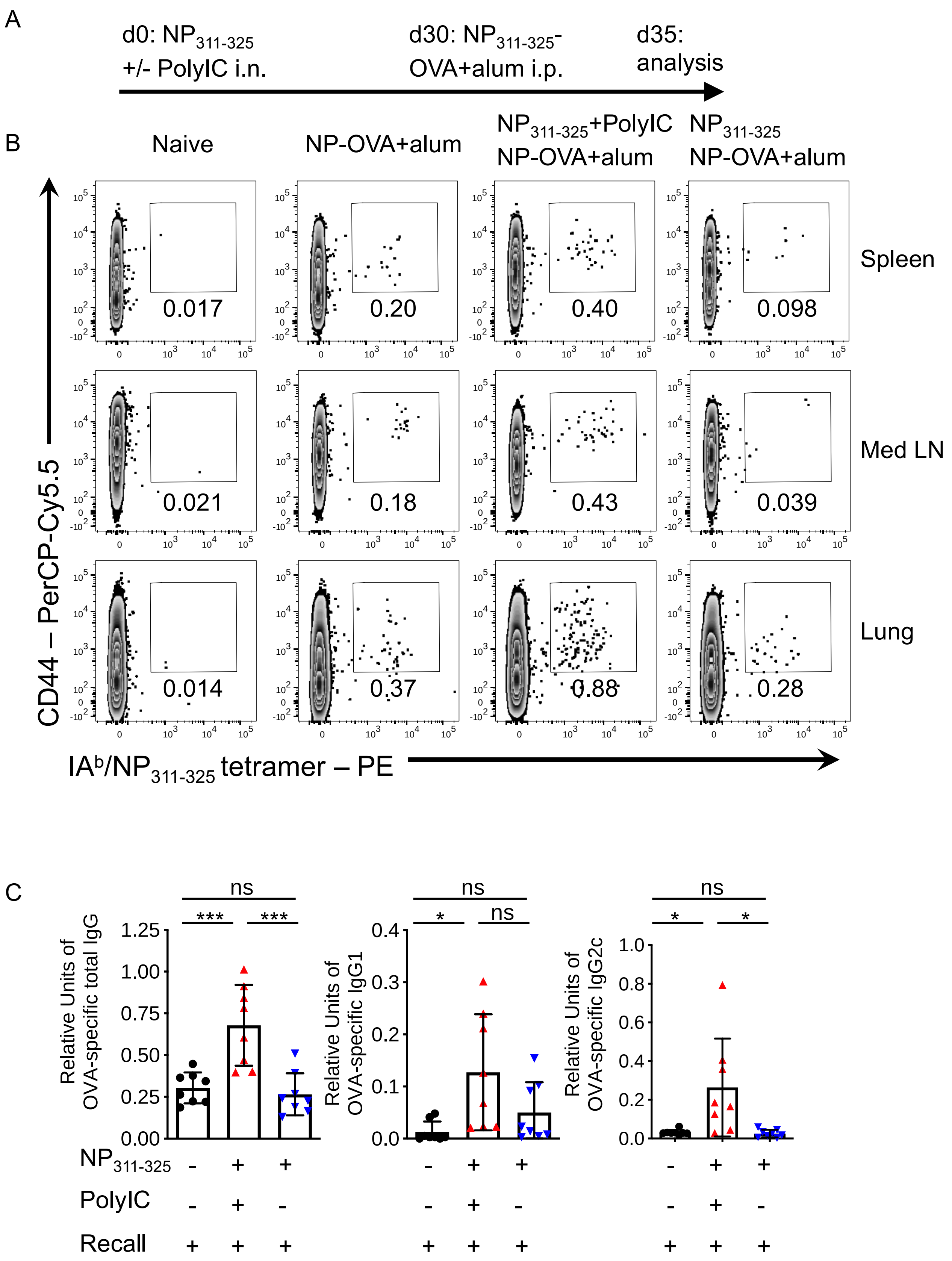

Supplementary Figure 4: Instillation of $NP_{311-325}$ peptide induces functional tolerance in naive animals

C57BL/6 mice were instilled with $NP_{311-325}$ peptide +/- PolyIC on day 0 and immunised with $NP_{311-325}$-OVA and alum i.p. 30 days later (A). The percentages of $IA_b/NP_{311-325}$ $CD44_{hi}$ CD4 T cells were examined 5 days after the recall immunisation (B). Cells are gated on live CD4+ lymphocytes that are negative for B220, CD8, F4/80, and MHCII+. The numbers on the graph show the percentages of the cells present in the gate within each plot. The levels of IgG, IgG1 and IgG2c anti-OVA antibodies was determined on day 5 in the serum (C). Data are from 2 experiments with 4mice/group. In C each point represents one mouse and the error bars are SD). All statistics calculated using a one-way ANOVA with multiple comparisons; ns = not significant, * = <0.05, ** = <0.01, *** = <0.001.

Supplementary Figure 5

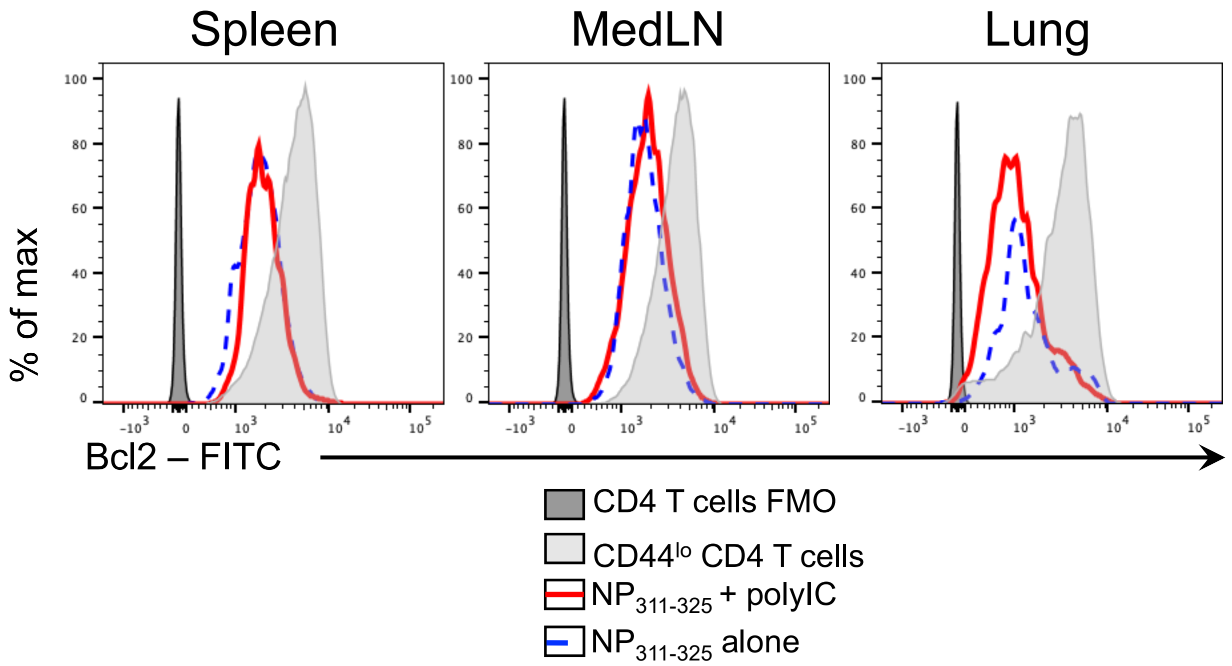

Supplementary Figure 5: Reactivated antigen specific memory CD4 T cells express similar levels of Bcl2 regardless of whether they were previously reactivated with peptide delivered with or without adjuvant

C57BL/6 mice were infected with IAV on day -30. On day 0, mice received $NP_{311-325}$ +/- PolyIC and immunised i.p with NP-OVA with alum on day 30. Cells examined 5 days later. The levels of Bcl2 within the cells was determined by flow cytometry. Cells are gated on CD44 high $IA_b/NP_{311-325}$ tetramer+ cells using the gating strategy shown in Supplementary Figure 1. Data are representative of animals from 4 experiments with 4-8 mice per experiment.

Supplementary Figure 6

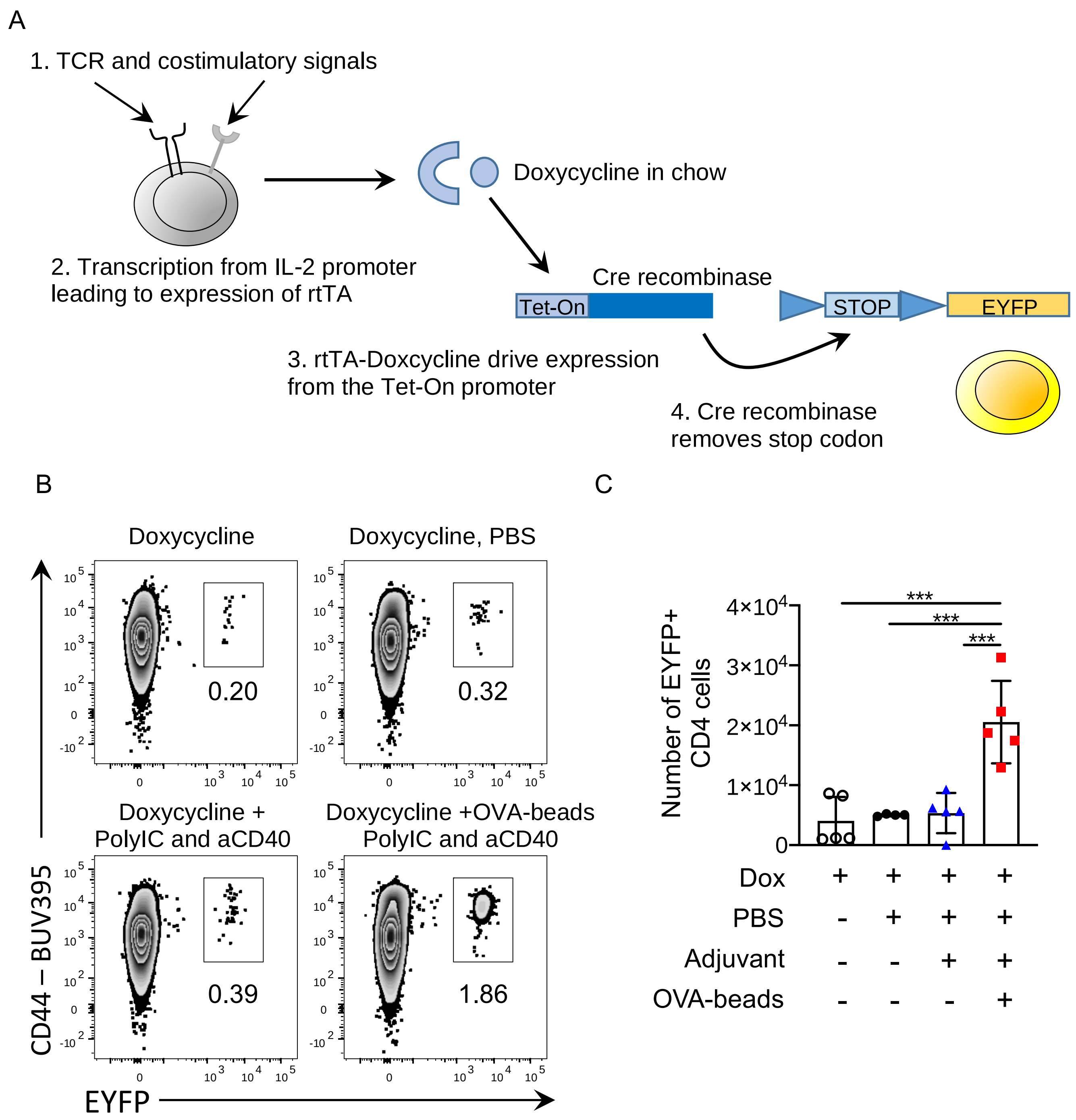

Supplementary Figure 6: TRACE mice enable identification of antigen-reactive CD4 T cells

In TRACE mice, activation through the TCR in the presence of costimulatory signals leads to the expression of rtTA driven by the interleukin 2 promoter. Only in the presence of doxycycline will rtTA be able to bind to the tet-ON promoter leading to the expression of Cre recombinase. Cre recombinase removes the stop codon at the Rosa locus allowing permanent expression of EYFP (A). In B, TRACE mice were given doxycycline chow from day minus 2 until day 5 and injected in the s.c. in the scruff on day 0 with nothing, PBS, anti-CD40 and PolyIC, or OVA protein conjugated to 20μm beads delivered with anti-CD40 and PolyIC. On day 9, the brachial and axillary lymph nodes were examined by flow cytometry. Cells are gated on live lymphocytes that were CD4+, and negative for B220, F4/80, MHCII and CD8. The numbers show the percentage of CD4+ cells that are in the indicated gates (A). In B, the numbers of EYFP+ CD4 T cells in the lymph nodes are shown with each symbol representing one mouse and error bars are SD. Statistics calculated using a one-way ANOVA with multiple comparisons; *** = <0.001.

Supplementary Table 1

Gene	ENSEMBL	log2FoldChange	pvalue	padj
Alox12	ENSMUSG00000000320	-24.38596644	1.23E-20	4.27E-17
Jchain	ENSMUSG00000067149	-24.20014489	2.67E-20	6.99E-17
Rbak	ENSMUSG00000061898	-23.94519388	1.09E-17	1.62E-14
Gm27166	ENSMUSG00000098519	-23.85260903	3.26E-19	6.83E-16
Kif4	ENSMUSG00000034311	-23.79895188	3.08E-17	4.02E-14
Ikzf4	ENSMUSG00000002578	-23.55188677	4.79E-17	5.57E-14
Adgrg3	ENSMUSG00000060470	-23.52967921	6.46E-17	6.76E-14
Gp1bb	ENSMUSG00000050761	-23.40514688	1.03E-16	9.82E-14
Tnfsf13b	ENSMUSG00000031497	-23.36580161	2.09E-16	1.56E-13
Gm10226	ENSMUSG00000067929	-23.36001221	1.78E-18	3.10E-15
2810021J22Rik	ENSMUSG00000020491	-23.32289945	2.06E-16	1.56E-13
Gm48693	ENSMUSG00000113157	-23.31146647	1.77E-16	1.54E-13
Trpm4	ENSMUSG00000038260	-23.21452431	2.39E-16	1.66E-13
Trgc1	ENSMUSG00000076749	-23.16312896	4.65E-16	2.86E-13
E430014B02Rik	ENSMUSG00000102973	-23.13782688	3.14E-16	2.05E-13
Engase	ENSMUSG00000033857	-23.05456996	6.37E-16	3.33E-13
5830444F18Rik	ENSMUSG00000102744	-23.02724878	5.37E-16	3.12E-13
Kdm8	ENSMUSG00000030752	-22.97619571	6.29E-16	3.33E-13
Hck	ENSMUSG00000003283	-22.87650475	1.14E-15	5.18E-13
Arhgef39	ENSMUSG00000051517	-22.85912417	1.29E-15	5.63E-13
Havcr2	ENSMUSG00000020399	-22.85288744	2.81E-15	1.18E-12
Tlr2	ENSMUSG00000027995	-22.77982105	3.68E-15	1.33E-12
Scg2	ENSMUSG00000050711	-22.75008302	8.41E-16	4.19E-13
Plk2	ENSMUSG00000021701	-22.72632542	3.36E-15	1.33E-12
Bckdhb	ENSMUSG00000032263	-22.72182153	3.62E-15	1.33E-12
Zfp109	ENSMUSG00000074283	-22.71884951	3.48E-15	1.33E-12
Gm44745	ENSMUSG00000108455	-22.66300493	5.68E-15	1.98E-12
Rgs9	ENSMUSG00000020599	-22.61504847	6.63E-15	2.24E-12
A930038B10Rik	ENSMUSG00000097310	-22.59861098	7.44E-15	2.43E-12
2900005J15Rik	ENSMUSG00000043833	-22.59847238	1.12E-15	5.18E-13
Slco4a1	ENSMUSG00000038963	-22.48471446	1.67E-14	5.29E-12
Cep152	ENSMUSG00000068394	-22.38149844	1.90E-14	5.83E-12

Supplementary Table 1: the top 33 gene genes expressed at lower levels in tolerogenic cells that are clustered are the far left of the Volcano plot in Figure 4B.

Supplementary Figure 7

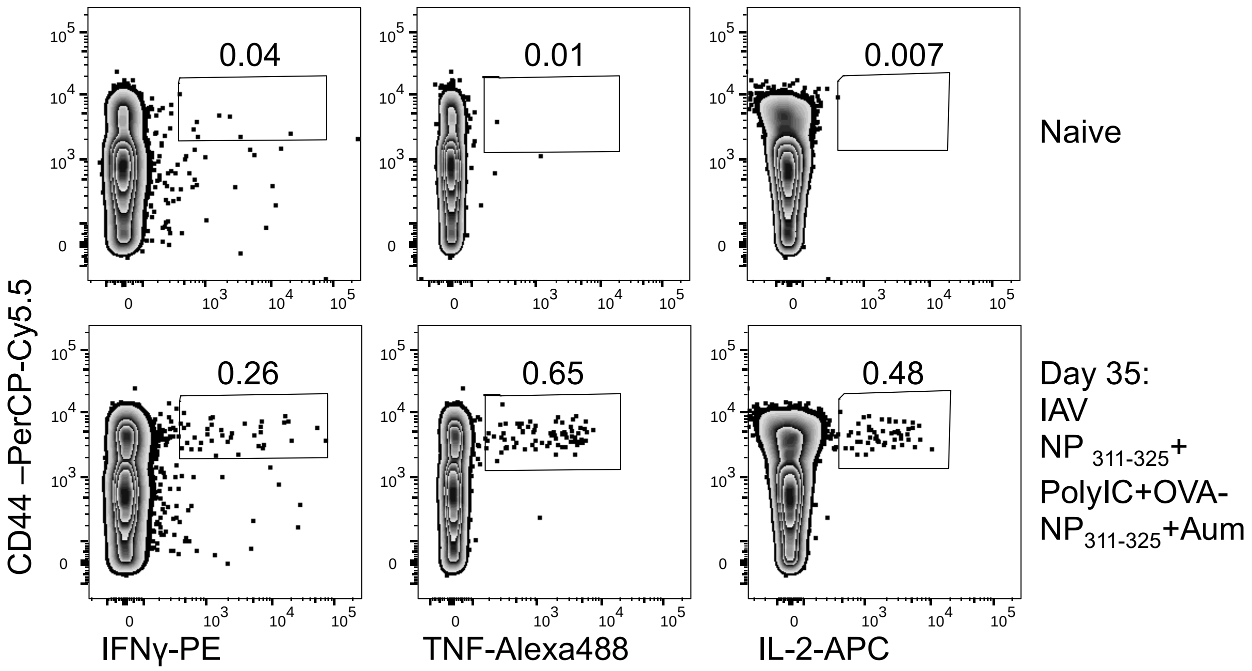

Supplementary Figure 7: Identification of $NP_{311\text{-}325}$ specific cytokine producing CD4 T cells

C57BL/6 mice were infected with IAV on day -30. On day 0, mice received $NP_{311\text{-}325}$ in the +/- PolyIC and some of these mice immunised i.p with NP-OVA with alum on day 30. On day 35, cells from the spleen were co-cultured with bmDCs loaded with $NP_{311\text{-}325}$ for 6 hours in the presence of Golgi Plug and the percentages of IFN-γ, TNF and IL-2 producing $CD44_{hi}$ CD4+ T cells examined. Cells are gated on live CD4+ lymphocytes as in gating strategy in SF1. Data are representative of 3 experiments (3-5mice/experiment).
